# In Silico Studies to Support Vaccine Development

**DOI:** 10.3390/pharmaceutics15020654

**Published:** 2023-02-15

**Authors:** Leonor Saldanha, Ülo Langel, Nuno Vale

**Affiliations:** 1 OncoPharma Research Group, Center for Health Technology and Services Research (CINTESIS), Rua Doutor Plácido da Costa, 4200-450 Porto, Portugal; 2 CINTESIS@RISE, Faculty of Medicine, University of Porto, Alameda Professor Hernâni Monteiro, 4200-319 Porto, Portugal; 3 Department of Community Medicine, Information and Health Decision Sciences (MEDCIDS), Faculty of Medicine, University of Porto, Rua Doutor Plácido da Costa, s/n, 4200-450 Porto, Portugal; 4 Institute of Technology, University of Tartu, Nooruse 1, 50411 Tartu, Estonia; 5 Department of Biochemistry and Biophysics, Stockholm University, 10691 Stockholm, Sweden

**Keywords:** vaccines, in silico, population pharmacokinetics, PopPK, PBPK, computational

## Abstract

The progress that has been made in computer science positioned in silico studies as an important and well-recognized methodology in the drug discovery and development process. It has numerous advantages in terms of costs and also plays a huge impact on the way the research is conducted since it can limit the use of animal models leading to more sustainable research. Currently, human trials are already being partly replaced by in silico trials. EMA and FDA are both endorsing these studies and have been providing webinars and guidance to support them. For instance, PBPK modeling studies are being used to gather data on drug interactions with other drugs and are also being used to support clinical and regulatory requirements for the pediatric population, pregnant women, and personalized medicine. This trend evokes the need to understand the role of in silico studies in vaccines, considering the importance that these products achieved during the pandemic and their promising hope in oncology. Vaccines are safer than other current oncology treatments. There is a huge variety of strategies for developing a cancer vaccine, and some of the points that should be considered when designing the vaccine technology are the following: delivery platforms (peptides, lipid-based carriers, polymers, dendritic cells, viral vectors, etc.), adjuvants (to boost and promote inflammation at the delivery site, facilitating immune cell recruitment and activation), choice of the targeted antigen, the timing of vaccination, the manipulation of the tumor environment, and the combination with other treatments that might cause additive or even synergistic anti-tumor effects. These and many other points should be put together to outline the best vaccine design. The aim of this article is to perform a review and comprehensive analysis of the role of in silico studies to support the development of and design of vaccines in the field of oncology and infectious diseases. The authors intend to perform a literature review of all the studies that have been conducted so far in preparing in silico models and methods to support the development of vaccines. From this point, it was possible to conclude that there are few in silico studies on vaccines. Despite this, an overview of how the existing work could support the design of vaccines is described.

## 1. Introduction

Vaccination plays a huge role in the prevention of many infections and has contributed to the eradication of certain diseases, saving millions of people’s lives [1]. More recently, it was possible to assist in the development of COVID-19 vaccines, which, within 1 year, were successfully developed and rapidly approved by health authorities in order to fight the pandemic. 

For many diseases, vaccines have been successful. However, there are still many pathogens for which there are no effective vaccines available, such as human immunodeficiency virus (HIV), tuberculosis (TB), respiratory syncytial virus (RSV), cytomegalovirus (CMV), herpes simplex virus (HSV), and Epstein–Barr virus (EBV) [2]. It is known that COVID-19 vaccines have endorsed innovative platforms in terms of technology, such as mRNA [3]. However, even with this technology, there are still pathogens for which successful vaccines have not yet been developed. The same applies to cancer vaccines. These have been investigated over the years, and only a few have been approved by FDA [4,5]. 

Overall and despite all the progress, there are certain challenges that are always raised during vaccine development: the relationship between the pathogen, the disease, and the population characteristics, and emerging infections, epidemics, or pandemics [6,7].

Strategies related to vaccine design need to be put in place to overcome these challenges. Innovative antigens, adjuvants, and delivery systems need to be outlined in order to achieve a successful vaccine for the diseases mentioned above. 

It is undeniable that computational science is nowadays a crucial tool within many fields. Its impact on drug discovery and development enhances many possibilities and has numerous advantages in terms of costs and the way the research is conducted. For instance, it can limit the use of animal models leading to more sustainable research [8,9]. In the future, there is also hope that in silico trials can replace human trials [10]. In fact, this is already a trend for certain populations. EMA and FDA have been endorsing these kinds of studies and providing webinars and guidance to support these trials. Physiologically Based Pharmacokinetic (PBPK) modeling studies, for instance, are being used to gather data on drug interactions with another drugs, pediatric population, pregnancy, and personalized medicine [11]. Overall, in silico studies allow a wide variety of simulations that can be helpful in drug design. From testing drug targets to predicting the drugs pharmacokinetics, pharmacodynamics, and so on. Regarding vaccines, it is also important to test immunologic properties and correlates of protection [12]. 

This review will highlight what has been performed so far in the field of vaccines for oncology and infectious diseases, using the in silico methodology and what studies have the potential to support the development of the vaccine. Through the exhaustive literature research, an overview of all in silico models created so far to support vaccine design will be described. 

## 2. Materials and Methods

The methodology used in this article can be divided into two phases. Phase 1 involved an exhaustive review of the literature in two major articles databases, PubMed and Web of Science. The research strategy for this review of the literature is outlined in Table 1 below. All the studies found were manually screened to verify their scope. The criteria established below in Table 2 were used to exclude articles during their assessment.

Phase 2 of the methodology was to compile all the in silico approaches prepared so far to aid vaccine development. Anything related to computer sciences that were endorsed in its scope for the support of vaccine development was considered (Table 3).

## 3. Results

### Results from Phase 1 (Literature Review and Screening)

**Figure 1:**
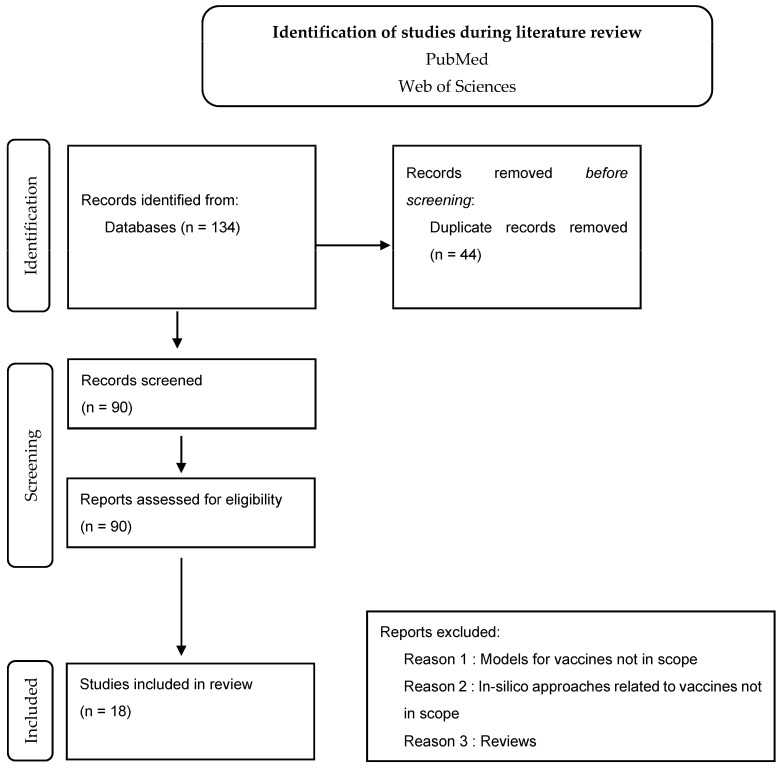
PRISMA flow-chart approach illustrates how the screening was performed.

While conducting Phase 1 of the methodology, the below-adapted PRISMA flow-chart approach illustrates how the screening was screened. The process can be checked in Figure 1.

## 4. Discussion

It is important to highlight that results reflect what is available in the public domain and that it is possible that some studies are being performed and sponsored by pharmaceutical companies and not yet being available. This is the main limitation of this review. Additionally, the term “in silico” is general. In this study, the authors considered “in silico” as any study involving computer science models to support drug and vaccine development and design.

According to the results from our study, there are seven studies using PBPK models to support vaccine development; three studies using PopPK; six studies using ABMs models; four ODEs models; 1 MP; and one study using different Computational Vaccine Design techniques. Some of these models combined two or more in silico approaches. For instance, for the Recombinant multi-epitope vaccine against influenza A virus model, a complex Computational Vaccine Design approach to predicting epitopes and selecting adjuvants, to evaluate physicochemical properties and solubility, to predict secondary structure properties, and finally, to immunogenicity and related immune. For these, many software and modeling techniques were combined. 

It is possible to verify that there are more models for infectious disease vaccines than for cancer vaccines by looking at the overall results. Additionally, PBPK modeling was the most used in silico approach to aid vaccine development, followed by ABM models, ODEs, and PopPK.

The reason behind PBPK models being the most common in terms of in silico approach might be due to the fact that this kind of modeling and simulation can be used to predict the drugs’ pharmacokinetics in humans using preclinical or clinical data. In parallel, population characteristics can be explored as well (i.e., age, ethnicity, or disease status). Furthermore, these models also play an important role in supporting the dose and dose regiment selection and also support predicting drug interactions. EMA and FDA are already currently accepting these kinds of studies to support regulatory decisions and have provided guidance to conduct PBPK modeling and simulations [11,31,32].

To date, multiple PBPK software has been created and used by various to support pharmaceutical drug development. Some of these platforms were discontinued, such as IDEATM (LION Bioscience, Inc.). However, others have remained in mainstream use and are currently being used by pharmaceutical companies and health authorities, such as EMA and FDA. Examples of the most commonly used software are GastroPlus (Simulation Plus, Inc.), Simcyp Simulator (Certara UK), and free tools such as PK-Sim [33].

ABM models were created to predict the immunogenicity of biological compounds and vaccines. This is because the immune system and its multiple agents and components are linked to complex interactions, and the ABM methodology allows these complex behaviors to emerge during simulation. This makes ABM perfect for performing biological simulations (i.e., for studying the complex and dynamic interactions within the biological environment) [34]. The Universal Immune System Simulator (UISS) platform is a type of ABM model and has been successfully applied to a large number of disease-modeling scenarios, including COVID-19, and can simulate, for instance, infection dynamics and its interactions with the host immune system, making it possible as well to predict the immunogenicity response of compounds [20,23].

ODEs represent models that are considered homogeneous, well-mixed systems and suited for traditional pharmacometrics analyses with sufficient data (population PK and PD models and PBPK models) or for simplistic theoretical PKPD models. They can also be used for quantitative clinical pharmacology models in order to study complex biological systems. Its limitation is related to extensive model assumptions, including parameter distributions. ABMs can provide more detailed insights into complex biological systems and are often complemented with ODEs in hybrid multi-scale models [35].

Population PK analyses are used to aid drug development and inform recommendations on therapeutic individualization (e.g., through tailored dosing). FDA states that adequate population PK data collection and analyses submitted in marketing applications, in some cases, have alleviated the need for postmarketing requirements and/or commitments [36]. PopPK models allow the study of variability in drug concentrations between individuals (healthy volunteers or patients). With this model, it is possible to assess the variability within the population and to account for the variability in terms of patient characteristics such as age, renal function, or disease state [37]. 

It is important to highlight that all methodologies have their strengths and weaknesses. It all depends on the purpose and context [35].

The most important information to retain with this review is that there are multiple in silico approaches that may complement each other and support pharmaceutical drug development. However, when we searched for in silico methodologies in vaccines, we only found 18 studies where models were prepared to support vaccine development. This means that research must continue in this field. 

Within the most common in silico approaches that were found for vaccine development, which is PBPK modeling, it can be verified that the most usual software, the GastroPlus and Simcyp Simulator, were not used. 

An interesting approach to future research would be to try to implement one of those existing PBPK models into one of the most common software and use their capacity to study different parameters to verify their applicability to vaccines. Performing simulations to test new adjuvants, improving formulation, targeting new antigens, and finding the best dose for different populations (considering age, ethnicity, or disease status on human pharmacokinetics) could be completed [14,31,38,39]. Sequentially, immunogenicity could be explored using UISS as an ABM through the simulation of the dynamics within the immune system. Currently, there are studies in the literature where UISS is applied to a broad range of diseases and not only to infections. An example of that is the application of UISS to multiple sclerosis pathogenesis, supporting the prediction of the disease and the treatment efficacy [40]. This reflects the flexibility of in silico software and highlights their capabilities to support the development of treatments for complex diseases with complex dynamics, such as cancer.

However, the results from this review show that there are not many models developed for vaccines in general and especially for cancer vaccines. This might be related to the complexity and challenges of vaccine development and the diseases themselves. 

Firstly, vaccine development itself is complex. It is known that most vaccine candidates fall in preclinical and early clinical development, and less than 1 in 15 candidates that enter Phase II will be approved. This is due to the lack of understanding of correlates of protection, not using appropriate animal models to predict responses in humans, complex dynamics and responses of the human immune system to antigens, and the synergies and impacts across the various components that can be combined in a vaccine [41].

Furthermore, in terms of vaccine efficacy, it is important to consider not only immediate protection but also long-term protection. Therefore, it is important to understand how to stimulate long-term memory, and this point is still not resolved. As an example, hepatitis B antigen vaccines produce lifelong protection, whereas for other vaccines, the protection is very short in terms of time. For this reason, it would be very important across the scientific community to develop in silico modeling to understand immune responses in humans [42]. 

Additionally, there are now more complex platforms for outlining the vaccine development strategies to overcome some issues of the standard vaccines, which are composed of inactivated pathogens. Advancements were made, and there are now new platforms related to DNA/RNA technologies, recombinant proteins, and the use of nanoparticles, for instance [43]. The reason for the development of such novel platforms is to aim for a more targeted immune response, to improve efficacy, and to provide long-term protection. There is also the hope that the new technologies will overcome the challenges of unmet needs for certain diseases, such as cancer and other complex diseases. However, as mentioned above, the limitation related to the lack of available data on these technologies may impact their development. This is why in silico studies might be a challenge in the field, but once they become more familiar with the area, they might support developers in important steps across the development of a vaccine, deciding which platform to use, adjuvants, formulations, and which dose and for which populations [43]. The vaccine design and formulation are extremely important in its overall efficacy. The adjuvants, for instance, support in improving the efficacy and the long-term immune response. However, it may also impact in the way the response is conducted [44,45].

Despite all the challenges, it is important to highlight the boost in vaccine development in terms of timelines. It took around 25 years to develop a vaccine for varicella, 5 years for Ebola, and 1 year for COVID-19 [46,47,48,49].

Therefore, in silico approaches, which can be used during all stages of development and discovery, can play an important role and contribute to the “boost” in vaccine development. PBPK studies, for instance, could help to predict the absorption, distribution, metabolism, and excretion (ADME) parameters of the candidates to improve efficacy. With this, it is also possible to save costs since this will reduce animal models and can also replace some trials [50,51]. 

To sum up, the complexity of vaccine development might be the reason why there are not many in silico models developed so far. The variability of the pathogen and tumors, the immunological responses, antigen selection, and memory of the responses are still the biggest challenges in the field. Due to genetic factors, age, disease status, and other factors, different responses may be expected [52]. 

Furthermore, when it comes to cancer vaccines, everything is even more complex. It is important to acknowledge that the pathological and immunological setting is different between cancer and infectious diseases. Acute inflammation is representative of infected tissues by pathogens, and this will trigger a potential development of protection in terms of immunity because the inflammation is obvious. Chronic inflammation environment is present with tumors, and these will repress anti-tumor immune responses, while tumor growth will be promoted in order to avoid the immune system. This means that lesions linked to tumors promote a not-so-obvious and, therefore, low-inflammatory environment. As a consequence, it is when the tissue is already very fragile due to tumor growth that the inflammation will become obvious [53]. Despite the complex environment in tumors, it is well known that the clinical translation of vaccines has been an issue. Most cancer vaccine clinical trials failed due to the selection of target antigens and the vaccines’ designs themselves, inducing very low-immunogenicity properties to have proper efficacy. The fact that there are only two therapeutic cancer vaccines approved by FDA and EMA, sipuleucel-T and talimogene laherparepvec [T-VEC], reflects all the complexity within the development of these platforms to treat cancer [54].

However, because so many studies have failed in the past, there is now more knowledge about these strategies, which are related to past failures. This means that lessons learned, together with new technological advancements, might be able to trigger a new era in cancer vaccine development, and in silico approaches will surely be part of it, as they already are for other complex diseases [28].

Considering the above, it means that further advancements are needed in the field of in silico studies for vaccines. Different types of models could be useful to overcome these issues: models to simulate host/pathogen/tumor interactions and models to simulate immune response [55,56,57].

Since regulatory authorities have clearly endorsed in silico models, such as PBPK, and even provided guides and frameworks to developers on how to integrate and achieve valuable data from them in drug development and discovery, considering that vaccines play an important role in the prevention and possibly in the treatment of certain diseases today, it is expected to see more models in the future [58,59,60].

In silico modeling, then, has the ability to save millions in terms of costs and could promote the selection of the best platforms, adjuvants (i.e., liposomes, nanoparticles), antigens (i.e., peptides) and dosages and dosage regimens in order to support the vaccines’ development and design [60,61,62,63,64].

## Figures and Tables

**Table 1:** Constructs, Strings, and identified articles from WoS and PubMed.

	Construct	Strings		No. of Articles Identified
Web of Science	vaccin* (All fields)	AND	“Physiological based pharmacokinetic” (All fields)	1
	vaccin* (All fields)	AND	“pbpk” (All fields)	18
	vaccin* (All fields)	AND	“Population Pharmacokinetics” (All fields)	50
	vaccin* (All fields)	AND	“poppk” (All fields)	2
	vaccin* (All fields)	AND	“in silico trial*” (All fields)	18
	Total articles			89
PubMed	vaccin* (All fields)	AND	“Physiological based pharmacokinetic” (All fields)	1
	vaccin* (All fields)	AND	“pbpk” (All fields)	14
	vaccin* (All fields)	AND	“Population Pharmacokinetics” (All fields)	15
	vaccin* (All fields)	AND	“poppk” (All fields)	2
	vaccin* (All fields)	AND	“in silico trial*” (All fields)	13
	Total articles			45

Note: PBPK—Physiological based pharmacokinetic and PopPK—Population Pharmacokinetics.

**Table 2:** Reasons to exclude identified articles.

Reason 1	Models for vaccines not in scope
Reason 2	In silico approaches related to vaccines not in scope
Reason 3	Review articles instead of vaccine model preparation

**Table 3:** Results from Phase 2 (Compilation of all in silico models to support vaccine development).

Product in Scope	Type of Model	Aim of Model	Comments	Software	**Authors; Year**
Formaldehyde-containing vaccines	PBPK	To assess the safety of residual formaldehyde in infant vaccines.	This model was used to predict formaldehyde disposition after an intramuscular injection.	CMATRIX	Robert J. Mitkus Maureen A.Hess Sorell L. Schwartz; 2013 [13]
Squalene-containing adjuvant vaccines	PBPK	To provide an estimation, quantitatively, of the squalene distribution in tissue following intramuscular injection.	This model was used to predict distribution after following intramuscular injection in humans.	Vensim PLE Plus (Ventana Systems, Inc., Harvard, MA, USA)	Million A. Tegenge Robert J. Mitkus; 2013 [14]
Nicotine vaccines	PBPK	To simulate and evaluate the efficacy of a nicotine vaccine.	The aim of the model is to predict the role of anti-nicotine antibodies on the nicotine disposition brain of humans and rats.	SimBiology	Kyle Saylor Chenming Zhang; 2016 [15]
α-tocopherol in emulsified-influenza vaccine adjuvant	PBPK	This model has two main goals. First, it is a PBPK model that will assess the in vivo fate of novel vaccine adjuvants; Secondly, it will predict the distribution of α-tocopherol in humans after a single dose of squalene-containing adjuvant vaccine	The aim of this model is to predict in vivo fate of α-tocopherol in adjuvanted influenza vaccine in humans after an intramuscular injection.	Vensim Professional^®^ (Ventana Systems, Inc., Harvard, MA, USA)	Million A.Tegenge Robert J. Mitkus; 2015 [16]
Cationic liposomal subunit antigen vaccine	PBPK	To predict human exposure to a cationic liposomal subunit antigen vaccine system.	The aim of the model is to predict the in-vivo fate of dimethyldioctadecylammonium bromide (DDA) and the immunostimulatory agent trehalose 6,6-dibehenate (TDB) (8:1 molar ratio) combined with the Ag85B-ESAT-6 (H1) in humans. Additionally, it aims to demonstrate what is the consequence of the formulation degradation and fraction escaping the depot site and what are the depot’s effects on the site of administration.	MATLAB (The MathWorks Inc., Natick, MA, USA, 2015)	Raj K. S. Badhan Swapnil Khadke Yvonne Perrie; 2017 [17]
Cancer vaccine	PBPK	To represent the distribution of certain molecules eluted through a 3D-printed implantable system named ‘NICHE’	The NICHE platform aims to study immunomodulation for cell therapeutics and cancer vaccines. It is a two-compartment model composed of a vascularized tissue reservoir and a surrounding refillable drug reservoir. The PBPK model was able to recapitulate the biodistribution of the molecules in scope, and together with NICH, they represent a flexible, adaptable platform to investigate local immunomodulation for biomedical applications.	Simbiology (MATLAB 2021b, Mathworks)	Simone Capuani et al.; 2022 [18]
Immune vaccines	PBPK through ordinary differential equations (ODEs)	To simulate and predict the distribution of different therapeutic agents and interactions with the immune system and its redistribution across lymphoid compartments. Furthermore, it allows the study of the infiltration into tumor tissues.	The aim of the model is to study the biodistribution of therapeutic agents and cells in blood and lymphatics, representing a PBPK novel model with tumor compartment properties enabling the study of key biological factors in the field.	Mathematical modeling	Javier Ruiz-Ramírez et al.; 2020 [19]
RUTI^®^ vaccine against tuberculosis	Agent-based model (ABM)	To predict the artificial immunity induced by RUTI^®^ vaccines using UISS.	The aim of the model is to predict the immune system’s complex dynamics by simulating mechanisms related to the infection and predicting how therapeutic strategies could face the infection.	Universal Immune System Simulator (UISS)	Marzio Pennisi et al.; 2019 [20]
	Agent-based model (ABM)	To assess and simulate the response of the combination of a standard anti-TB therapy strategy with a potential therapeutic vaccine, such as RUTI.	The model simulates the disease activities and their interaction within the immune system. Additionally, it allows the prediction of the efficacy of the combination of isoniazid and RUTI vaccine in a certain digital population cohort.	Universal Immune System Simulator (UISS)	Giulia Russo et al.; 2020 [21]
Specific tuberculosis vaccines: RUTI and ID93/GLA-SE	Agent-Based Model (ABM)	This is an EU—funded STriTuVaD project computational platform. It allows the prediction of immunity provided by RUTI and ID93/GLA-SE.	A multi-scale (cellular and molecular level), multi-compartment, polyclonal agent-based simulator that predicts the ability to predict the immunity induced by RUTI and ID93/GLA-SE (both tuberculosis vaccines).	Universal Immune System Simulator (UISS)	Giulia Russo et al.; 2019 [22]
COVID-19 candidate vaccines	Agent-based model (ABM)	This model aids the testing and designing of therapeutics against SARS-CoV-2. Its intention is to allow a boost in vaccine development to predict any failures and minimize side effects.	A model to predict the efficacy of therapy against COVID-19.	Universal Immune System Simulator (UISS)	Giulia Russo et al.; 2020 [23]
Yellow fever vaccine	Ordinary differential equations (ODE)	These mathematical models allow the study of primary and secondary responses to the yellow fever virus.	A model integrated by ordinary differential equations, which aim is to study responses to the yellow fever virus in five populations: yellow fever virus, three types of B cells (naive, active, and memory), and antibodies.	Mathematical models	Larissa de L. e Silva et al.; 2020 [24]
Squalene-containing emulsion vaccine adjuvants	PopPK	Estimating PK parameters are important to the study of squalene properties after intramuscular administration of influenza vaccines.	The aim of the study is to simulate PK parameters that are properties after intramuscular injection. Results aim to contribute to the knowledge of an informed benefit-risk assessment of a vaccine containing squalene as an adjuvant.	NONMEM^®^ 7.3, Hanover, MD	Million A.Tegenge et al.; 2016 [25]
HIV vaccine	PopPK	To demonstrate the pharmacokinetics properties and predict HIV-1 neutralization.	This model aims to assess and predict VRC01 serum concentration and serum neutralization titer to panels of HIV-1 isolates in order to validate a potential biomarker to support an HIV vaccine development.	NONMEM software system (version 7·4, ICON Development Solutions).	Yunda Huang et al.; 2021 [26]
HIV vaccine	PopPK	This model supports the estimation of individual-specific VRC01 concentrations as correlates of protection (CoP). It assesses the association between the value of VRC01 concentration and the instantaneous rate of HIV infection.	To simulate population characteristics and study visits data, R version 3.5.1 R Core Team (2016) was used. With the NONMEM software system (Version 7.4, ICON Development Solutions), it was possible to model concentration data.	R version 3.5.1 R Core Team (2016) NONMEM software system (Version 7.4, ICON Development Solutions)	Lily Zhang et al.; 2021 [27]
Cancer vaccines	In silico model population (MP)	This model will support the prediction of clinical outcomes for cancer vaccines.	With the in silico modeling, it was possible to predict the frequency of vaccine-specific HLA-binding epitopes in order to calculate the immune response rate (IRR) for the model population.	Immune Epitope Database (IEDB)	Orsolya Lőrincz et al.; 2021 [28]
Designing therapeutics for vaccines	Agent-based model (ABM)	To provide a description of the cellular behavior of the immune system and dynamics.	These three model pieces are linked to cross-information in all scales. It is a mathematical and also multi-scale model (including both cellular- and molecular-level events).	ABM is constructed using the C++ programming language, Boost libraries (distributed under the Boost Software License: http://www.boost.org), and the Qt framework for visualization (distributed under GPL: http://www.qt.digia.com). T; Simulations performed on Nyx/Flux computing cluster available at the Center for Advanced Computing at the University of Michigan	Jennifer J. Linderman, Nicholas A. Cilfone, Elsje Pienaar, Chang Gong, Denise E. Kirschner; 2015 [29]
	Ordinary differential equations (ODEs)	To record events related to receptor–ligand binding, trafficking, and intracellular signaling.			
	Relevant partial differential equations	To describe the diffusion of certain ligands, cytokines, and other components.			
Recombinant multi-epitope vaccine against influenza A virus	Computational vaccine design	Retrieving influenza protein sequences and multiple alignments	The NCBI database and Jalview software were used to expose the amino acid sequences and to perform the multiple alignments, respectively.	NCBI database and Jalview software	Avisa Malek et al.; 2021 [30]
		B-cell epitopes prediction	This is an important step in synthetic peptide vaccine development. These epitopes should be capable of evoking antibodies in order to neutralize the pathogen.	SVMTriP IEDB Analysis	
		CTL epitopes prediction	NetCTL 1.2 server was used to identify MHC class I epitopes.	NetCTL 1.2 server	
		CD4 T-cell epitopes prediction	NetMHCIIpan 4.0 was used to identify MHC class 2 epitopes.	NetMHCIIpan–4.0	
		Antigenicity and allergenicity prediction of CTL, CD4 T-cell, and B-cell epitopes	In order to verify the antigenicity of the peptides, the VaxiJen v2.0 was used. In parallel, to evaluate their allergenicity, the software AllerTOP v2.0 was used. The toxicity of peptides was assessed with ToxinPred.	VaxiJen v2.0 AllerTOP v2.0 TxinPred	
		Human population coverage analysis	To verify and assess human population coverage, IEDB was used.	IEDB	
		Recombinant multi-epitope vaccine	Analyses were made of three vaccine adjuvants in order to select the candidate for the final vaccine formulation.	BCEPS web server	
		Evaluation of physicochemical properties and solubility	To reveal the physicochemical properties of the vaccine, ProtParam was used. The solubility was assessed with the Protein-sol server.	ProtParam Protein-sol server	
		Secondary structure prediction of the recombinant vaccine	The secondary structure of the final formulation and its properties was predicted with the RaptorX Property web server.	PSIPRED 4.0 web server RaptorX Property web server	
		Codon adaption and in silico cloning of the recombinant vaccine	Reverse translation and codon optimization for candidates were conducted with JAVA Codon Adaptation Tool (JCat).	JAVA Codon Adaptation Tool (JCat)	
	Agent-based model (ABM)	In silico trial simulation of the immune system	Immune response and immunogenicity were assessed with UISS.	Universal Immune System Simulator (UISS)	

## Data Availability

Not applicable.
